# Diarrheal Diseases Hospitalization in Yemen before and after Rotavirus Vaccination

**DOI:** 10.1155/2016/8485417

**Published:** 2016-06-29

**Authors:** Mohammed Amood AL-Kamarany, Lina Al-Areqi, Abulatif Mujally, Fawzya Alkarshy, Arwa Nasser, Aisha O. Jumaan

**Affiliations:** ^1^Department of Pharmacy Practice, Faculty of Clinical Pharmacy and Tropical Medicine Center, Hodeidah University, P.O. Box 3114, Hodeidah, Yemen; ^2^Program of Health and Drug, Tihama Foundation for Drug Studies and Research, Hodeidah, Yemen; ^3^The Yemeni-Swedish Hospital, Taiz, Yemen; ^4^Independent Consultant, Seattle, WA, USA

## Abstract

The study aims to assess the impact of rotavirus vaccine introduction on diarrheal diseases hospitalization and to identify the rotavirus genotypes most prevalent before and after vaccine introduction among children ≤ 5 years of age. Rotarix*™* ® rotavirus vaccine is currently licensed for infants in Yemen and was introduced in 2012. The vaccination course consists of two doses. The first dose is administrated at 6 weeks of age and the second dose is completed by 10 weeks. Based on a longitudinal observational study, we assessed the impact of vaccination on rotavirus hospitalization before and after vaccination among children ≤ 5 years of age at the Yemeni-Swedish Hospital (YSH) in Taiz, Yemen. Prevaccination covered January 2009–July 2012 during which 2335 fecal samples were collected from children ≤ 5 years old. Postvaccination covered January 2013–December 2014 during which 1114 fecal samples were collected. Rotavirus was detected by Enzyme Linkage Immunosorbent Assay (ELISA). The incidence of* rotavirus* hospitalization decreased from 43.79% in 2009 to 10.54% in 2014. Hospitalization due to rotavirus diarrhea was reduced by 75.93%. Vaccine coverage increased from 23% in 2012 to 72% in 2014. Also, the results showed that the most predominant genotypes in prevaccination period were G2P[4] (55.0%), followed by G1P[8] (15.0%), while in postvaccination period G1P[8] (31%) was the predominant genotype, followed by G9P[8] (27.5%). In conclusion, rotavirus vaccination in Yemen resulted in sharp reduction in diarrheal hospitalization. A successful rotavirus vaccination program in Yemen will rely upon efficient vaccine delivery systems and sustained vaccine efficacy against diverse and evolving rotavirus strains.

## 1. Introduction

Rotaviruses are the leading cause of severe gastroenteritis in infants and young children < 5 years of age worldwide and they are the cause of approximately half a million deaths each year [1–3], leading to an enormous disease burden; every minute a child dies because of rotavirus infection. A previous study reported that rotavirus is the most common cause of diarrhea hospitalization (45.2%) in infants and young children aged ≤ five years in Taiz, Yemen [4]. G2P[4] was the predominant genotype (55.0%), followed by G1P[8] (15.0%), and 4% each of G1G2P[8] and G1G2P[4]P[8], 7% of undetermined G2, and 15% of other genotypes [4].

Intense efforts to develop rotavirus vaccines started in the 1980s, and by 2006 two vaccines were licensed in many countries [5–7]. Yemen introduced rotavirus vaccine (*Rotarix*) in July 2012. Vaccine is administered in two doses. The first dose is administrated at 6 weeks of age and the second dose is completed by 10 weeks [8]. This study examines the impact of the rotavirus vaccination program on hospitalization in same area and, in addition, evaluates the update of the rotavirus genotypes after vaccination.

## 2. Methods

### 2.1. Study Area

Taiz is one of the largest governorates in Yemen, with an area of 10.000 km^2^ and a population of about 2.5 million inhabitants, living in 23 districts. The total population of children < five years is 540,000 according to the last national census conducted to in 2004 [9].

### 2.2. Study Design

A longitudinal observational study was conducted by recruiting children ≤ 5 years of age hospitalized for diarrhea at the Yemeni-Swedish Hospital (YSH) in Taiz. The study was conducted from January 2009 until December 2014 at the only public hospital which admits children and provides maternal health care in Taiz city. The incidence of rotavirus hospitalization before vaccination was estimated from January 2009 to July 2012 during which 2335 fecal samples were collected and the incidence of hospitalization after vaccination was estimated from August 2012 to December 2014 during which 1330 fecal samples were collected.

### 2.3. Ethical Issue

Mothers or child's guardians received a simple explanation of the aim of the study and were asked to participate. If they agreed, the sample was collected and an interview was conducted. Confidentiality of the collected data was achieved by keeping data record in a locked room with limited access to the research team only.

### 2.4. Data Collection

Within two days after hospitalization, at least 4–8 mg of stool was directly collected and stored in a sterile plastic container. Samples were kept at 2–8°C for a maximum of 8 days until they were transported to the laboratory where they were stored at −20°C prior to analysis. Clinical information was obtained from the child's mother or guardian. Information included the child's sex, age at admission, symptoms, hydration status, height, weight, and length of hospital stay.

### 2.5. Rotavirus Analysis by Enzyme Linked Immunosorbent Assay (ELISA)

Rotavirus infection status was ascertained by ELISA (IDEIA Kit, Dako Ltd., Cambridgeshire, UK) using a polyclonal antibody prepared against the common antigen presented on rotavirus VP6 (a major group specific protein). These antibodies were used in a solid phase sandwich type ELISA using a microplate containing 96 wells [10].

### 2.6. Detection of Rotavirus* Genotypes* by Real Time-Polymerase Chain Reaction (PCR)

Rotavirus genotypes were detected by Reverse Transcription Polymerase Chain Reaction (RT-PCR) on 29 cases of rotavirus-positive samples. Two amplification RT-PCR were carried out for identification of rotavirus gene (P-Genotyping). For determination of the G types, the viral RNA was extracted according to the instructions of QI amplification viral RNA mini kit (Qiagen, USA) and specific primers were used. The extracted RNA was kept at −20°C until use [11, 12].

### 2.7. Agarose Gel Electrophoresis

All PCR products were also examined by gel electrophoresis in 2% agarose gel and the rotavirus genotypes were determined by the molecular weight of the amplicons. On the other hand, gel separating nucleic acid requires staining in order to be visualized under ultraviolet illumination. This stain contained ethidium bromide [11, 12].

### 2.8. Statistical Methods

Demographic and laboratory results data were entered and analyzed using Excel Software 2010. Descriptive analysis and Chi-square test were used to make comparisons among categorical variables. For all statistical analyses, a *p* value of less than 0.05 was considered statistically significant. Due to political unrest in 2011, reporting was disrupted for all health outcomes including our study. Therefore, we excluded the 2011 data. Furthermore, since vaccine was introduced in mid-2012, we also excluded 2012 from the analysis.

## 3. Results

### 3.1. Subject Characteristics

A total of 3143 children ≤ 5 years were diagnosed with diarrhea and admitted to the hospital from January 2009 to December 2014. Of those, 2334 were admitted from January 2009 to July 2012 to the prevaccination program in Yemen and 1330 were admitted from August 2012 to December 2014 to the postvaccination program. The age range of patients was from 1 to 60 months. More male patients than female patients were admitted (61% versus 39%), respectively. Children aged 6–8 and 9–12 months experienced the highest infections.

### 3.2. Rotavirus Diarrhea before Vaccination

**Figure 1:**
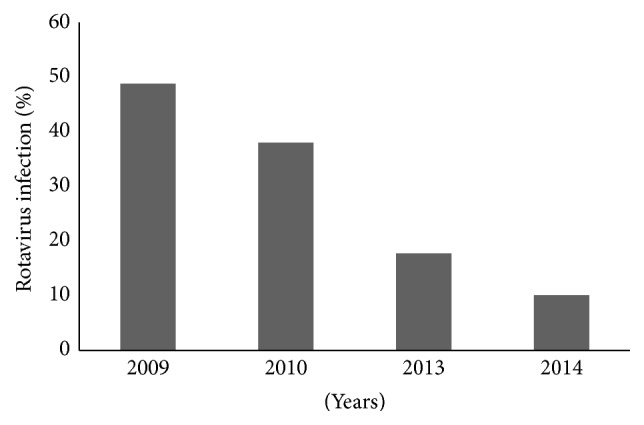
Incidence of rotavirus hospitalization that tested positive before and after vaccination in Taiz Yemen, 2009–2014.

Fecal samples were collected from 1776 children ≤ 5 years of age diagnosed with diarrhea during the period from January 2009 to December 2010 in prevaccination stage (Figure 1). All children had diarrhea for a period of 1-2 days before hospitalization. Rotavirus diarrhea was detected in 41.5% during 2009-2010. The rotavirus incidence was higher in 2009, 43.8% compared to 37.4% in 2010 (Table 1). More males than females were affected in both years with about 63.5 of the infections occurring in males in 2009 and 60.18 in 2010. Children aged 6–8 months in 2009 and those aged 9–12 months had the highest infection percent (27.3%, 30.1%), respectively.

### 3.3. Rotavirus Diarrhea after Vaccination

In postvaccination stage, fecal samples were collected from 1114 children ≤ 5 years of age diagnosed with diarrhea during the period from January 2013 to December 2014 (Figure 1). The incidence of rotavirus diarrhea was higher in 2013, 17.9% compared to 10.5% in 2014 (Table 1). Overall rotavirus hospitalization was reduced to 14.1% after vaccination in 2013-2014. The incidence of rotavirus diarrhea in infants and young children was reduced by 75.9% between 2009 and 2014 which was statistically significant with *p* value (*p* < 0.05). The reduction was highest among males at 79.5% and at 69.8% among females. The reduction was also observed among all age groups and was highest among children less than 2 years of age (Table 1).

### 3.4. Seasonality of Rotavirus Diarrhea before and after Vaccination

**Figure 2:**
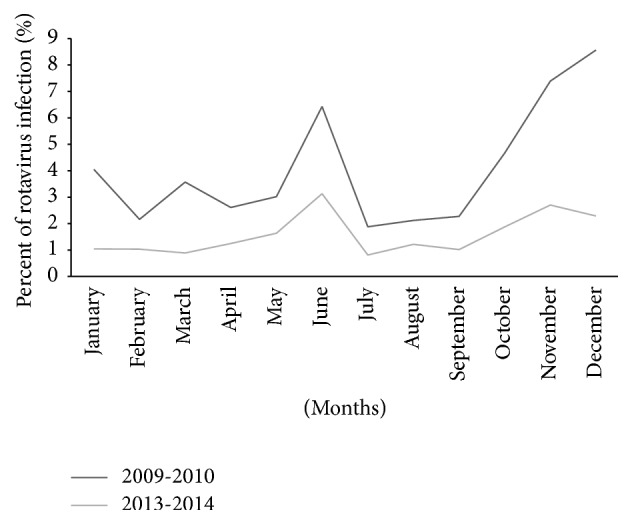
Seasonality of hospitalization for rotavirus infection in Yemen, Taiz, before vaccination (2009-2010) and after vaccination (2013-2014).

The seasonal distribution of rotavirus during prevaccination years showed a higher frequency during the winter, namely, December (140 cases, 8.7%), and lower frequency during the summer season, namely, July (31 cases, 1.9%). Similar findings were also observed after vaccination, with a higher frequency during the winter, namely, November (31 cases, 2.8%), and lower frequency during the spring season, namely, March (6 cases, 0.9%). However, the seasonality was less pronounced after vaccination (Figure 2).

### 3.5. Genotypes of Rotavirus Infection before and after Vaccination

In prevaccination period G and P genotypes of rotavirus were detected in 73 cases by RT-PCR assay. G2P[4] was the predominant genotype (55%), followed by G1P[8] (15%). In addition, rotavirus genotypes in postvaccination period were detected in 29 cases, and G1P[8] was the predominant genotype (31%), followed by G9P[8] at 27.5% (Table 2).

## 4. Discussion

Rotavirus is the most common cause of severe diarrhea in children worldwide and diarrhea deaths in children in developing countries. The present study showed that rotavirus was the most common cause of diarrhea (40.0%) in infants and young children ≤ five years in Taiz in prevaccination period.

Our results showed a significant decrease in rotavirus hospitalization after vaccination; the decrease occurred in all age groups, especially those less than 2 years of age, and for males and females. This decrease corresponded to national* rotavirus* vaccine coverage of 71% and 72% in 2013 and 2014, respectively [8]. Our results support findings from earlier published studies; studies found rotavirus diarrhea to be at 46% and 40% in Jeddah, Saudi Arabia, and Kuwait, respectively [13, 14].

In Taiz, the vaccination program against rotavirus diarrhea improved child health from this common burden affecting all children. The results showed that 40.60% of the specimens were rotavirus-positive in prevaccination period and was reduced to 14.09% in postvaccination period. The incidence of hospitalization due to rotavirus diarrhea in infants and young children was reduced by 75.95%. This result supports other studies; Grimwood and Lambert described that “in high and middle income countries, rotavirus vaccines confer 85–100% protection against severe disease, while in low income regions of Africa and Asia, protection is less, at 46–77%.” In our study, of the 1114 cases who were admitted for diarrhea in 2013 and 2014, 422 patients were vaccinated; of these, only 89 (21.1%) had rotavirus diarrhea, compared to 333 (78.9%) who did not have rotavirus diarrhea; this difference was statistically significant (*p* < 0.05). Postlicensure effectiveness studies show that rotavirus vaccines not only reduce rotavirus activity in infancy but they also decrease rates of rotavirus diarrhea in older and unimmunized children [15].

The incidence of rotavirus hospitalization in children before vaccination was high. Therefore, the vaccine had high impact resulting in the decline of rotavirus hospitalization among children aged ≤ 5 years. The vaccine had high impact resulting in the decline of rotavirus hospitalization among children aged ≤ 5 years. Our results support findings from previous published studies; rotavirus hospitalization was reported to decline by 69%–80% in El Salvador [16] and 65%–83% in Belgium [17]. Also, lower decline of rotavirus hospitalization was recorded in developed countries, namely, Mexico (11%–40%) [18] and Panama (22%–37%) [19]. In contrast, higher decline of rotavirus hospitalization was recorded in Australia (89%–94%) [20].

Two live oral rotavirus vaccines have been licensed in many countries. Each vaccine has proven highly effective in preventing severe rotavirus diarrhea in children and safe from the possible complication of intussusceptions. The vaccines are the only public health prevention strategy employed in Yemen during our study period to control rotavirus disease and vaccine coverage increased from 23% in 2012 to 72% in 2014 [8]. Therefore, we believe that the reduction we observed is due to the introduction of rotavirus vaccination. Efficacy of these vaccines has ranged from 80% to 98% in industrialized countries, including Latin America, and 39% to 77% in developing countries, such as Africa and Asia [21].

During the period of the present study rotavirus diarrhea hospitalization occurred throughout the year, but the greatest number of rotavirus diarrhea hospitalization was identified in November and December, supporting findings from earlier studies that reported higher infections in the cold, dry season in Tunisia [22] and in some African countries where rotavirus infection was recorded, such as Morocco [23, 24], Algeria [25], and Egypt [25]. In contrast, in one study, the higher frequency of rotavirus infection was recorded in May and June in Taiz, Yemen, in 2007 and 2008 [4]; similarly, in Jordan, rotavirus was more frequent during the summer months, from June to August [26]. The seasonality was less pronounced after vaccination.

The virus is classified into G and P serotypes and further into P genotypes based on differences in the surface-exposed proteins VP7 and VP4, respectively, [15]. The peak incidence of rotavirus diarrhea occurs between 0 and 24 months of age, similar to our findings. In Yemen, G2P[4] (55.0%) and G1P[8] (15.0%) are responsible for most diseases [4] in prevaccination period and these rates can be reduced or prevented by accelerated development and introduction of rotavirus vaccines into global immunization program has been a high priority for many international agencies, including WHO and the Global Alliance for Vaccines and Immunizations. In postvaccination period, rotavirus genotypes, namely, G1P[8] (31%) and G9P[8] (27.5%), were more predominant. The genotyping of rotavirus variation after vaccination was compared with previous studies. G1P[8] which is the predominant genotype in our study is similar to the strain in Turkey and Australia [20, 26].

The variation of rotavirus genotypes was found through the two periods that were attributed to seasonal variation. There is great diversity of rotavirus strains in children with severe gastroenteritis in Taiz. Even though cross-protection with vaccine-induced immunity occurs, continued strain surveillance is recommended after the introduction of rotavirus vaccine in the national immunization program. There is concern that antigenically distinct novel or rare strains may be selected and spread, decreasing vaccine efficacy [27, 28]. In addition, the next generation of rotavirus vaccines, including parenterally administered, nonreplicating rotavirus vaccines, are in various stages of preclinical and clinical development; such understanding is critical to maximizing the public health impact of the current vaccines and also to the development of the next generation of rotavirus vaccines, which are needed [28].

## 5. Conclusion

A successful rotavirus vaccination program in Yemen will rely upon efficient vaccine delivery systems and sustained vaccine efficacy against diverse and evolving rotavirus strains.

## Figures and Tables

**Table 1:** Number and percent of rotavirus diarrhea hospitalization before vaccination (*N*: 1776) and after vaccination (*N*: 1114).

Age (months)	2009 *n*: 870		2010 *n*: 906		2013 *n*: 535		2014 *n*: 579		*p* value^*∗*^
	Male	Female	Male	Female	Male	Female	Male	Female	
0–2	13	5	11	5	7	0	1	2	*p* < 0.05
3–5	39	18	27	20	3	4	4	6	*p* < 0.05
6–8	61	43	51	29	20	14	3	1	*p* < 0.05
9–12	49	27	58	44	17	11	8	8	*p* < 0.05
13–17	52	32	25	19	4	2	9	7	*p* < 0.05
18–23	21	9	19	8	5	2	2	2	*p* < 0.05
24–59	7	5	13	10	6	1	6	2	*p* < 0.05

Total number (*n*)	242	139	204	135	62	34	33	28	
Percent (%)	63.5%	36.5%	60.2%	39.5%	64.6%	35.4%	54.1%	45.9%	

^*∗*^ *p* value is comparing 2009-2010 to 2013-2014.

**Table 2:** Genotypes of rotavirus infection before and after vaccination.

	Rotavirus genotype before vaccination			Rotavirus genotype after vaccination	
	*n* = 73			*n* = 29	
	Rotavirus			Rotavirus	
	Percent (%)	Number (*n*)		Percent (%)	Number (*n*)
G2P[4]	55.0%	40	G1P[8]	31%	9
G1P[8]	15.0%	11	G9P[8]	27.5%	8
G1G2P[8]	4%	3	G2G9P[4]P[8]	10.3%	3
G1G2P[4]P[8]	4%	3	G9P[4]P[8]	10.3%	3
G2 untypeable	7%	5	G9P[4]	6.8%	2
Other genotypes	15%	11	G9 untypeable	3.4%	1
—	—		Untypeable P[8]	10.34%	3

Total (*n*)	100%	73	—	100%	29
